# Circular RNA circEYA3 promotes the radiation resistance of hepatocellular carcinoma via the IGF2BP2/*DTX3L* axis

**DOI:** 10.1186/s12935-023-03168-2

**Published:** 2023-12-02

**Authors:** Pan Hu, Letao Lin, Tao Huang, Zhenyu Li, Meigui Xiao, Huanqing Guo, Guanyu Chen, Dengyao Liu, Miaola Ke, Hongbo Shan, Fujun Zhang, Yanling Zhang

**Affiliations:** 1 Department of Minimally Invasive Intervention, State Key Laboratory of Oncology in South China, Guangdong Provincial Clinical Research Center for Cancer, Sun Yat-Sen University Cancer Center, 651 Dongfeng Road East, Guangzhou, 510060 Guangdong People’s Republic of China; 2 School of Laboratory Medicine and Biotechnology, Southern Medical University, 1023 South Shatai Road, Guangzhou, Guangdong 510515 People’s Republic of China; 3 Department of Experimental Research, State Key Laboratory of Oncology in South China, Guangdong Provincial Clinical Research Center for Cancer, Sun Yat-Sen University Cancer Center, Guangzhou,, 510060 People’s Republic of China; 4 Department of Blood Transfusion, State Key Laboratory of Oncology in South China, Guangdong Provincial Clinical Research Center for Cancer, Sun Yat-Sen University Cancer Center, Guangzhou,, 510060 People’s Republic of China; 5 Department of Endoscopy, State Key Laboratory of Oncology in South China, Guangdong Provincial Clinical Research Center for Cancer, Sun Yat-Sen University Cancer Center, 651 Dongfeng Road East, Guangzhou, 510060 Guangdong China

**Keywords:** Circular RNA, RNA-binding protein, ^125^I brachytherapy, Radiation resistance

## Abstract

**Background:**

Hepatocellular carcinoma (HCC) has a high incidence and mortality rate despite various treatment options, including ^125^I seed implantation. However, recurrence and radiation resistance remain challenging issues. Hsa_circ_0007895 (circEYA3)—derived from exons 2–6 of *EYA3*–facilitates the proliferation and progression of pancreatic ductal adenocarcinoma. However, the role of circEYA3 in HCC ^125^I radiation resistance remains unclear. Thus, we aimed to investigate the functions and underlying molecular mechanisms of circEYA3 in HCC under ^125^I and X-ray irradiation conditions.

**Methods:**

CircEYA3 was identified by RNA-seq in patients with HCC before and after ^125^I seed implantation treatment, followed by fluorescence in situ hybridization and RNase R assays. The radiosensitivity of HCC cell lines irradiated with ^125^I seeds or external irradiation were evaluated using the Cell Counting Kit 8, flow cytometry, γH2A.X immunofluorescence and comet assays. RNA pull-down and RNA immunoprecipitation assays were performed to explore the interactions between circEYA3 and IGF2BP2. *DTX3L* mRNA was identified by RNA-seq in PLC/PRF/5 cells with overexpressed circEYA3. The corresponding in vitro results were verified using a mouse xenograft model.

**Results:**

CircEYA3 decreased the radiosensitivity of HCC cells both in vitro and in vivo. Notably, using a circRNA pulldown assay and RNA-binding protein immunoprecipitation, we identified IGF2BP2 as a novel and robust interacting protein of circEYA3. Mechanistically, circEYA3 binds to IGF2BP2 and enhances its ability to stabilize *DTX3L* mRNA, thereby specifically alleviating radiation-induced DNA damage in HCC cells.

**Conclusions:**

Our findings demonstrate that circEYA3 increases the radioresistance of HCC to ^125^I seeds and external irradiation via the IGF2BP2/*DTX3L* axis. Thus, circEYA3 might be a predictive indicator and intervention option for ^125^I brachytherapy or external radiotherapy in HCC.

**Supplementary Information:**

The online version contains supplementary material available at 10.1186/s12935-023-03168-2.

## Background

Primary liver cancer (PLC) incidence has been increasing, with approximately 906,000 new cases and more than 830,000 deaths worldwide in 2020, among which hepatocellular carcinoma (HCC) accounts for 75–85% of PLC cases [1]. The onset of HCC is intricately linked with various risk factors, most notably cirrhosis induced by either hepatitis B virus (HBV) or hepatitis C virus (HCV) infection, in addition to other contributors like alcohol abuse, aflatoxin B1 intake and metabolic syndrome [2]. The high incidence and mortality rate of HCC can largely be attributed to its insidious nature. Among patients diagnosed primarily based on symptomatic presentation, approximately 70–80% have already missed the opportunity for complete tumor resection [3]. Localized HCC is treated with radical resection surgery, ablation therapy, or transcatheter arterial chemoembolization; however, in the context of advanced HCC, the utilization of the aforementioned treatment modalities often yields only modest extensions in patient survival, while the combined application of multiple therapeutic methods may be accompanied by substantial and deleterious side effects [4]. Iodine-125 (^125^I) seed implantation therapy has continuous and low-dose-rate radiation, which cause DNA damage in tumor cells mainly by releasing large amounts of X-rays and γ-rays, leading to G2/M arrest, mitotic inhibition, and apoptosis induction, significantly decreasing tumor cell proliferation, invasion, and metastasis [5–7]. Owing to its advantages of accurate targeting toward tumors and minimal damage to the surrounding tissues, 125I seed implantation therapy is applied in recurrent HCC [8]. However, resistance of HCC to ^125^I brachytherapy has limited its clinical effects [9]. Thus, the mechanisms of radiation resistance in HCC need to be further explored, and novel therapeutic targets and predictive biomarkers are needed to improve the survival rate of patients with HCC.

Circular RNA (circRNA) is a class of coding or non-coding RNA formed by covalent binding (non-canonical splicing or back-splicing) of linear pre-mRNA 5′ and 3′ ends [10, 11]. However, the functions of most circRNAs remain unknown. In the past, circRNAs were considered by-products of RNA splicing with low abundance and poor cell conservation. Recent studies have gradually overturned this perspective [12]. CircRNAs are involved in diverse HCC behaviors, including tumorigenesis, invasion, metastasis, and drug resistance [13–15]. Zhu et al. identified circ-LARP1B, a novel circRNA, which enhanced the radiation resistance of HCC cells against external radiotherapy in vitro and in vivo [16]. However, the role of circRNAs in radiation resistance during ^125^I brachytherapy remains unclear.

Here, we identified a ^125^I radiosensitivity-related circRNA, circEYA3, derived from the eyes absent homolog 3 (*EYA3*), with a circBase [17] ID of hsa_circ_0007895. We further explored the functions and underlying molecular mechanisms of circEYA3 in HCC under ^125^I and X-ray irradiation conditions in vivo and in vitro. Functionally, the overexpression of circEYA3 reduced DNA damage and enhanced radioresistance of HCC cells to ^125^I seeds and external irradiation. CircEYA3, which was prominently localized in the cytoplasm, decreased tumor radiosensitivity by stabilizing deltex E3 ubiquitin ligase 3L (*DTX3L*) mRNA via its interaction with insulin-like growth factor 2 mRNA-binding protein 2 (IGF2BP2).

## Materials and methods

### Patient blood specimens and cell lines

Six pairs of blood samples were obtained from patients with HCC before and after ^125^I seed implantation therapy for exosome extraction and RNA sequencing (RNA-seq). This study was approved by the Ethics Committee of the Sun Yat-sen University Cancer Center. Written informed consent was obtained from all participants of the study.

For the ^125^I seed implantation procedure, computed tomography (CT)-guided percutaneous puncture implantation was used. Briefly, preoperative localization was performed with a 16-multidetector-row CT scanner (Brilliance CT BigBore; Phillip Medical Systems, the Netherlands) guidance. After determining the entry point and path of the needles, 18-gauge needles were inserted into the tumors at intervals of at least 1.0 cm according to the preoperative plan. Precautions were taken to avoid the puncture of large blood vessels and important organs. After the needles were placed at the predetermined position, the ^125^I seeds were implanted at 0.5 cm intervals. Bleeding, pneumothorax and other complications were excluded by rescanning CT. The clinical information of patients undergoing RNA sequencing has been compiled in Additional file 1: Table S1.

PLC/PRF/5 and HepG2 HCC cell lines were purchased from Cellcook (Guangdong, China), cultured in minimum essential medium (Gibco, Grand Island, NY, USA) supplemented with 10% fetal bovine serum (ExCell Biological Technology, Suzhou, China), 1% penicillin–streptomycin (Thermo Fisher Scientific, Waltham, MA, USA), and 1% non-essential amino acids (ELGBIO Biotechnology, Guangdong, China) and maintained in a humidified cell incubator at 37 ℃ and 5% CO_2_. All cells were tested using a MycoBlue Mycoplasma Detector (Vazyme Biotech, Nanjing, China) and confirmed to be mycoplasma-negative before use in the experiments.

### Exosome extraction

The exosomes were extracted by ultracentrifugation. Briefly, plasma samples were collected from patients and centrifuged for 30 min at 500 × *g*. Supernatants were centrifuged at different speeds (2,000 × *g*, 10,000 × *g*, and 100,000 × *g*) to remove dead cells and cell fragments and obtain exosomes and interfering proteins. Subsequently, the pellet was resuspended in phosphate-buffered saline (PBS) and centrifuged at 100,000 × *g* for 70 min. This experiment was performed at 4 ℃. Finally, PBS was used to resuspend the pellet before storage at –80℃ for further experiments.

### RNA-seq

The RNA-seq experiments were conducted by Epibiotech Co., Ltd. Briefly, RNA from plasma exosomes was extracted using the exoRNeasy Serum/Plasma Maxi kit (Qiagen, Hilden, Germany). Subsequently, the RNA was reverse-transcribed to cDNA using Evo M-MLV RT Master Mix (Accurate Biology, Changsha, China). The reverse transcription process involved incubation at 37 °C for 20 min, followed by denaturation at 85 °C for 30 s. RNA sequencing was conducted utilizing the Illumina HiSeq X10/Hiseq 4000 platform. The expression values of circRNAs were predicted based on the anticipated circRNA sequences. This prediction was achieved by tallying the reads associated with circRNAs, encompassing both TopHat mapping and TopHat-fusion mapping reads. The expression levels were then quantified as RPM (Reads Per Million mapped reads). A heat map was drawn based on the expression values obtained.

### Quantitative real-time PCR (qRT-PCR)

An RNA-Quick Purification Kit (Yishan Biotechnology, Shanghai, China) was used to extract total RNA from the cell lines. After measuring the RNA concentration using a NanoDrop 3000 spectrometer (Thermo Fisher Scientific, Waltham, MA, USA), RNA was reverse-transcribed to cDNA using Evo M-MLV RT Master Mix (Accurate Biology). The reverse transcription was carried out at 37 °C for 20 min, followed by denaturation at 85 °C for 30 s. qRT-PCR analysis was performed using the LightCycler 480 System (Roche, Basel, Switzerland) using iTaq Universal SYBR Green Supermix (Bio-Rad Laboratories, Hercules, CA, USA). The specific temperatures and times for each step were as follows: initial denaturation at 95 °C for 5 min; amplification with 45 cycles of denaturation at 95 °C for 10 s, annealing at 60 °C for 10 s, and extension at 72 °C for 10 s; melt curve analysis at 95 °C for 5 s, 65 °C for 1 min, and 97 °C continuous; and cooling at 40 °C. Glyceraldehyde-3-phosphate dehydrogenase (GAPDH) and U6 was used as endogenous control to calculate relative mRNA or circRNA expression with the 2^–ΔΔCT^ method. All PCR primer sequences are listed in Additional file 1: Table S2.

### *circRNA fluorescence *in situ* hybridization (FISH)*

Cy3-labeled circEYA3 probes were obtained from Geneseed Biotech (Guangdong, China). FISH assay was performed using the HepG2 cell line. Briefly, cells were fixed with 4% paraformaldehyde and permeabilized with 0.5% Triton X-100. After drying, the pre-denatured (100 μM) probes were added to the culture well and incubated overnight at 37℃ in a humidified atmosphere. The slides were then rinsed thrice with saline sodium citrate buffer (Yaneng Bioscience, Shenzhen, China), followed by DAPI staining. Images were captured using the FV1000 confocal laser scanning microscope (Olympus, Tokyo, Japan). FISH probe sequence is as follows: 5' TTCACAATCAAAAGGAGGTAGTC 3'.

### Nuclear-cytoplasmic fractionation

Nuclear and cytoplasmic RNA isolation was performed using Cytoplasmic & Nuclear RNA Purification Kit (AmyJet Scientific, Wuhan, China). Briefly, the cells were lysed with Lysis Buffer J and centrifuged at 14,000 × *g* for 10 min. The supernatant was transferred to a RNase-free centrifuge tube, and Buffer SK was added to both the supernatant and the pellet. The resulting mixture was then introduced into a centrifugal column and centrifuged at 3500 × *g* for 1 min. Following three washes of the centrifugal column with Wash Solution, RNA was eluted using Elution Buffer for subsequent qRT-PCR experiments.

### RNase R treatment

Total RNA (5 μg) was extracted as mentioned above and treated with 3 U/μg RNase R (Geneseed Biotech, Guangzhou, China) and incubated for 30 min at room temperature. Then, the treated RNAs were incubated at 70 ℃ for 10 min to inactivate the enzyme. Relative RNA abundance was detected by qRT-PCR using circEYA3 and *EYA3* linear mRNA primers.

### Plasmid vector construction and transient transfection

The overexpression plasmids of circEYA3 were constructed by Geneseed Biotech. Overexpression plasmids for IGF2BP2 and short hairpin RNAs (shRNAs) against IGF2BP2 were obtained from Genechem (Shanghai, China). These sequences are listed in Additional file 1: Table S2. For transient transfection, Lipofectamine 3000 Transfection Reagent (Thermo Fisher Scientific) was used according to the manufacturer’s instructions. Briefly, the transfection mixture, plasmids, and Opti-MEM I Reagent (Thermo Fisher Scientific) were added to 6-well plates when cell confluence reached 70–80%. The transfection medium was replaced 6–8 h later with normal medium.

### Animal experiments

Male 4-week-old nude mice were purchased from Vital River (Beijing, China). All animals were treated in accordance with the guidelines of the Committee on Animals of Sun Yat-sen University. A subcutaneous xenograft tumor model was constructed as previously described. Briefly, 1 × 10^6^ cells were subcutaneously injected into the right flank of the mice. When the average tumor diameter reached 5 ± 1 mm, ^125^I seeds were implanted. Under ultrasound guidance, a dedicated seed implantation needle was precisely positioned within the tumors. Subsequently, a ^125^I seed applicator was employed to facilitate the implantation of seeds (0.8 mCi per seed) at the desired locations of the tumors. Seven days after the implantation of ^125^I seeds, the circEYA3 overexpression plasmid (15 μg/mouse/injection) was injected into multiple sites within the tumor every 4 days for a total of three injections. Tumor tissues were collected on day 21.

### Immunofluorescence (IF)

Briefly, cells were fixed and permeabilized as described above. The cells were washed twice with PBS and blocked with 5% bovine serum albumin. γH2A.X antibody (#9718, Abcam, Cambridge, MA, USA) and the corresponding secondary antibody were incubated with cells sequentially, followed by staining with DAPI (KeyGEN BioTECH, Nanjing, China). Finally, fluorescence images were captured using FV1000 confocal laser scanning microscope (Olympus).

### Single cell gel electrophoresis (SCGE)

A CometAssay SCGE Kit (R&D Systems, Tustin, CA, USA) was used. Briefly, 2 h after irradiation or continuous ^125^I exposure for 2 days, the cells were resuspended in PBS and mixed with low-melting-point agarose gel. The mixture was then spread onto the solidified normal-melting point agarose gel and allowed to stay for 30 min at 4 ℃ in the dark. After immersion in the lysis buffer, the slides were subjected to electrophoresis at 25 V for 30 min. Next, 40 μL propidium iodide (PI) was added for staining and observed using the ECLIPS Ti-2 inverted microscope (Nikon, Tokyo, Japan).

### Cell proliferation assays

Cell viability was measured every 24 h for 5 consecutive days using the Cell Counting Kit-8 (CCK-8, Yishan Biotechmology, Shanghai, China). Transfected cells were seeded into 96-well plates at a density of 5 × 10^3^/well with or without irradiation exposure. After incubation with the CCK-8 assay solution for 2 h, the absorbance of the cells in the microplate reader (BioTek Instruments, Winooski, VT, USA) was determined at 450 nm. Cell viability was normalized to the absorbance on day 1.

### Flow cytometry

To measure the apoptosis rate, the Annexin V-AF647/PI Apoptosis Kit (Yishan Biotechnology) was used. Cells seeded in 6-well plates were digested with 0.25% trypsin, centrifuged, and resuspended in binding buffer. Then, 10 μL Annexin V-AF647 and 5 μL PI were added to the cell suspension followed by incubation for 5 min in the dark. Flow cytometry was then performed using the CytoFLEX flow cytometer (Beckman Coulter, Pasadena, CA, USA).

### RNA pulldown assay

RNA pulldown assay was performed to identify circEYA3-binding proteins, according to the manufacturer’s protocol. Briefly, by incubating the cell lysates with streptavidin-coated magnetic beads, the biotin-coupled RNA complex was pulled down, and the combined protein was eluted from the filled beads. Subsequently, western blotting (WB) and mass spectrometry analyses were performed.

### RNA immunoprecipitation (RIP)

RIP assay was conducted using the IEMed-K303 RIP Kit (IEMed, Guangzhou, China) according to the manufacturer’s instructions. Briefly, the cells were lysed with lysis buffer and incubated with an anti-IGF2BP2 antibody or negative control immunoglobulin G overnight. Magnetic beads were then added, and the cells were resuspended with Proteinase K to remove proteins. RNA was purified and analyzed using qRT-PCR.

### WB

Proteins were extracted from the cell lines using radioimmunoprecipitation lysis buffer containing phosphatase and protease inhibitors. After the protein concentration was measured using the BCA Protein Quantitation Assay Kit (KeyGEN), equal amounts of total proteins were separated by sodium dodecyl sulfate–polyacrylamide gel electrophoresis (ACE Biotechnology, Nanjing, China) and transferred to polyvinylidene difluoride membranes (Millipore, Burlington, MA, USA). The membranes were incubated with the corresponding primary and secondary antibodies, followed by detection with enhanced chemiluminescence (GBCBIO, Guangdong China) using the ChemiDoc MP Imaging System (Bio-Rad Laboratories), with GAPDH as a control. The primary antibodies used were anti-GAPDH (1:5000; Proteintech, Shanghai, China), anti-IGF2BP2 (1:5000; Proteintech), anti-DTX3L (1:600; Proteintech), anti-γH2A.X (1:1000, Cell Signaling Technology, MA, USA), anti-caspase-3 (1:1000, Cell Signaling Technology), and anti-cleaved caspase-3 (1:1000, Cell Signaling Technology).

### Statistical analysis

GraphPad Prism 8 (GraphPad Software, San Diego, CA, USA), SPSS version 23.0 (IBM, Armonk, NY, USA), and R version 4.1.0 (R Foundation for Statistical Computing, Vienna, Austria) were used for statistical analysis. If the data adheres to a normal distribution, it is expressed as mean ± standard deviation and subjected to analysis using the Student’s t-test. In cases where normal distribution is not met, representation is done as median with interquartile range, and analysis is performed using the Wilcoxon test. All tests were conducted as two-sided tests and *p* ≤ 0.05 was considered statistically significant. All data were generated by at least triplicate independent experiments.

## Results

### ***Identification of ***^***125***^***I brachytherapy-associated circRNAs and characterization of circEYA3***

**Fig. 1:**
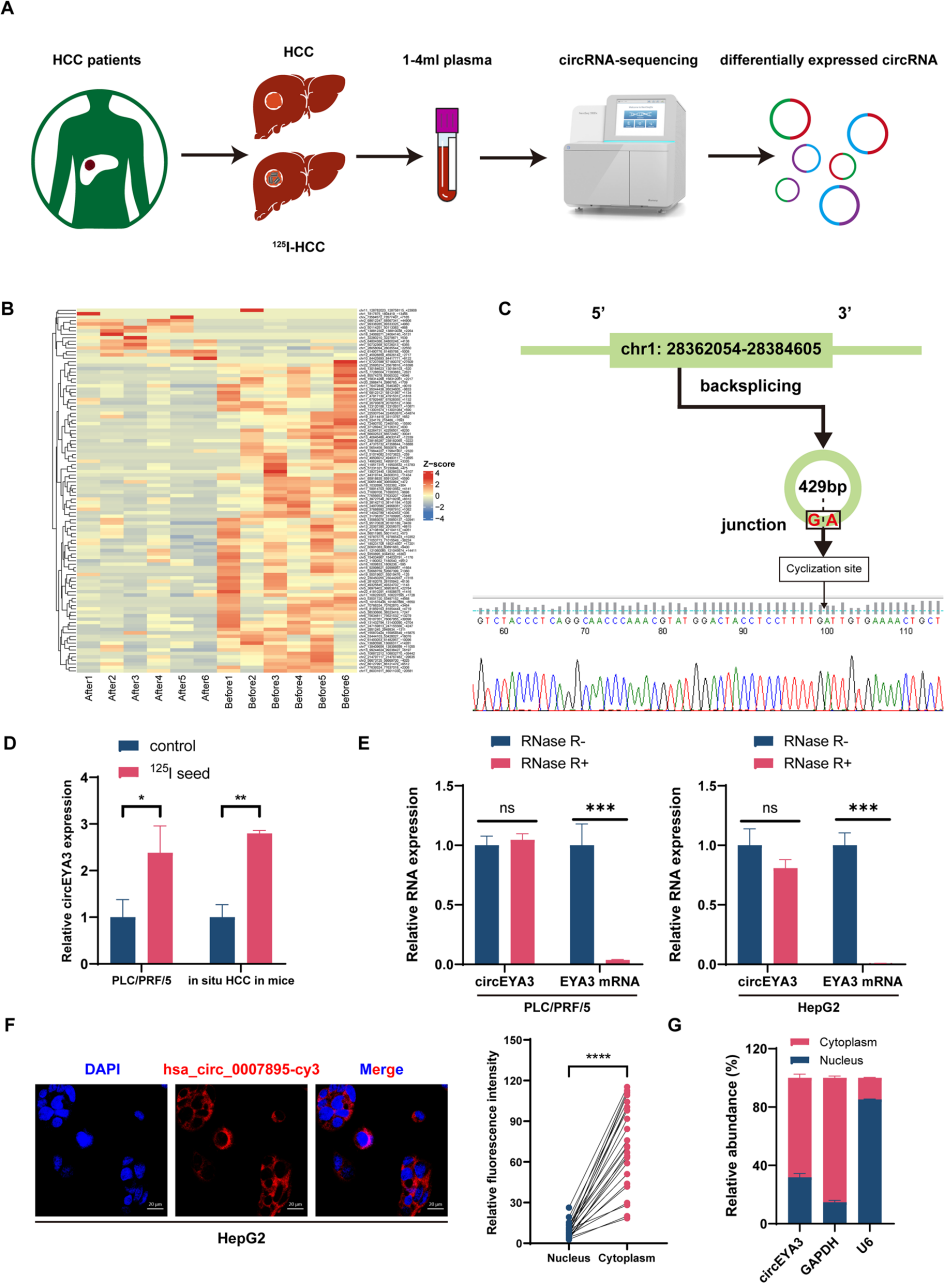
Screening and characterization of circEYA3. **A** Flowchart of differentially expressed circular RNA (circRNA) screening of patients with hepatocellular carcinoma (HCC) after implantation of ^125^I seeds. **B** The heat map of differentially expressed circRNA before and after ^125^I seed implantation. **C** Schematic showing the origin of circEYA3. Sanger sequencing indicated the existence of circEYA3 and the back-splicing junction sites in exons 2 and 6 of *EYA3*. **D** The relative levels of circEYA3 in PLC/PRF/5 cell line and in situ HCC in mice with or without.^125^I irradiation were detected by quantitative real-time PCR (qRT-PCR). **E** qRT-PCR was performed to detect the circEYA3 and *EYA3* mRNA levels after RNase R treatment in PLC/PRF/5 and HepG2 cells, respectively. **F** Fluorescence in situ hybridization (FISH) was carried out to determine the distribution of circEYA3 in HCC cells. Red fluorescence (Cy3-labeled probe) indicates circEYA3, whereas the nuclei were stained with DAPI (blue). Scale bar: 20 μm. **G** Nuclear-cytoplasmic fractionation experiments were conducted to determine the subcellular localization of circEYA3

To screen ^125^I brachytherapy-related circRNAs, we collected blood samples from patients with HCC before and after ^125^I seed implantation treatment (Fig. 1A). Subsequently, exosomes extracted from the plasma were subjected to circRNA sequencing and bioinformatics analyses. A total of 106 differentially expressed circRNAs were identified using the parameters *p* < 0.05 and |log_2_fold change|> 1 (Fig. 1B). By querying the functions of the parental genes of these circRNAs, we screened the top 10 significant circRNAs related to radiosensitivity and verified their expression in tumor tissue samples from patients with HCC (Additional file 1: Table S3). Among these, circARPC1AA, circLRCH3, circASAP2, and circUSP4 could not be amplified due to junction site constraints. The expression levels of the remaining 6 circRNAs in HCC were observed. Consequently, hsa_circ_0007895, exhibiting the highest expression, was chosen for subsequent experiments. Additionally, we validated the expression of hsa_circ_0007895 in HCC cells and in situ HCC mouse models before and after ^125^I seed irradiation. The results indicated an upregulation of hsa_circ_0007895 post-irradiation (Fig. 1D).

The circBase database (http://circrna.org/) shows that hsa_circ_0007895 is an exonic circRNA formed by the cyclization of exons 2–6 of *EYA3*, with a length of 429 bp. We termed hsa_circ_0007895 as “circEYA3” and then determined whether it was a bona fide circRNA. Sanger sequencing was performed to verify the back-splicing junction site (Fig. 1C). In addition, the RNA extracted from PLC/PRF/5 and HepG2 cell lines was exposed to RNase R, a 3′ to 5′ exoribonuclease, to assess the stability of circEYA3. The results indicated that compared with its linear counterpart, circEYA3 was more resistant to RNase R digestion, proving that circEYA3 had a circular structure (Fig. 1E). Furthermore, the RNA FISH assay showed that circEYA3 was predominantly localized in the cytoplasm of HepG2 cells (Fig. 1F). Additionally, nuclear-cytoplasmic fractionation experiments further indicated that circEYA3 predominantly localizes in the cytoplasm, consistent with the findings from FISH experiments (Fig. 1G). Collectively, these results demonstrated that circEYA3 was a stable and abundant circular transcript related to ^125^I treatment in HCC.

### ***circEYA3 alleviates ***^***125***^***I-induced tumor growth inhibition ***in vivo*** and DNA damage in HCC cells ***in vitro

**Fig. 2:**
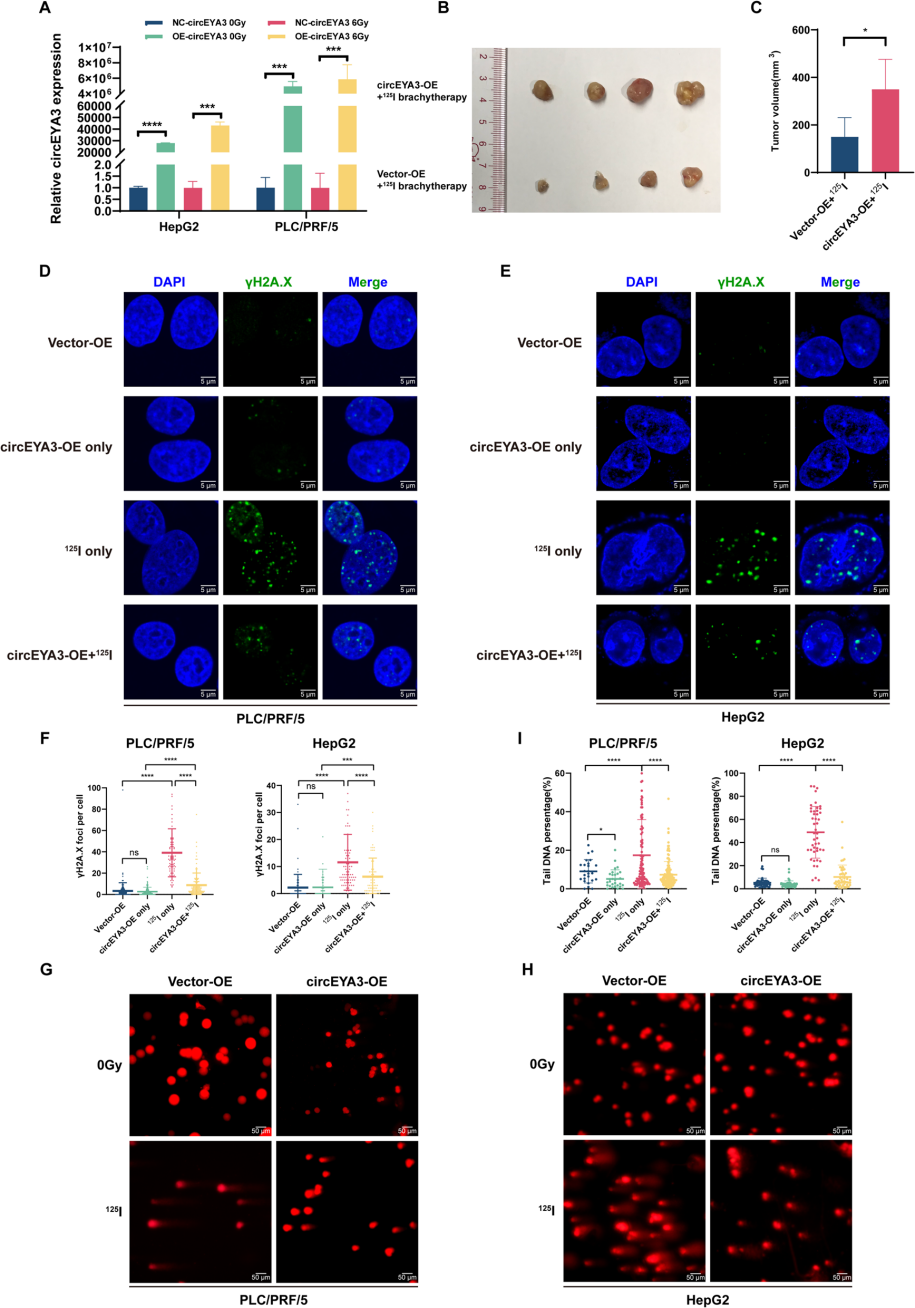
circEYA3 alleviates ^125^I-induced tumor growth inhibition in vivo and DNA damage of HCC in vitro. **A** qRT-PCR was employed to detect the relative levels of circEYA3 in PLC/PRF/5 and HepG2 cells after transfection with the circEYA3 overexpression plasmid or the control plasmid. **B**, **C** Image and bar chart of tumors in mice that received or did not receive ^125^I brachytherapy. **D**, **E**, **F** Immunofluorescence experiments were conducted to observe γH2A.X foci in PLC/PRF/5 and HepG2 cells with or without ^125^I treatment. **G**, **H**, **I** Single cell gel electrophoresis assay (SCGE) or comet assays were performed to observe the DNA damage caused by.^125^I irradiation with or without overexpression of circEYA3

To evaluate the role of circEYA3 in HCC ^125^I radiosensitivity, circEYA3 overexpression vectors were designed, constructed, and validated by qRT-PCR in the PLC/PRF/5 and HepG2 cell lines. The results indicated that circEYA3 was significantly overexpressed with or without irradiation in both cell lines (Fig. 2A). In vivo experiments showed that after ^125^I brachytherapy, tumor growth in the circEYA3 overexpression group was significantly inhibited compared with that in the control group (Fig. 2B, C).

To further explore whether circEYA3 increases the radioresistance of HCC during ^125^I brachytherapy, we conducted γH2A.X IF and SCGE assays in vitro. Histone H2A.X phosphorylation on serine four residues from the carboxyl-terminus (producing γH2A.X) is a sensitive marker of DNA double-strand breaks (DSBs) [18]. The IF assay indicated that ^125^I radiation caused a sharp increase in γH2A.X foci in the nuclei. However, under irradiation conditions, overexpression of circEYA3 decreased γH2AX foci in both cell lines (Fig. 2D-F). The SCGE assay involves the electrophoresis of DNA strand breaks or fragments to the anode in an alkaline electrophoresis solution, resulting in the formation of tails. The results showed that the increase in tail DNA percentage caused by ^125^I irradiation was partially reversed by circEYA3 overexpression (Fig. 2G-I).

### circEYA3 protects HCC cells from DNA damage, proliferation inhibition, and apoptosis induced by external irradiation

**Fig. 3:**
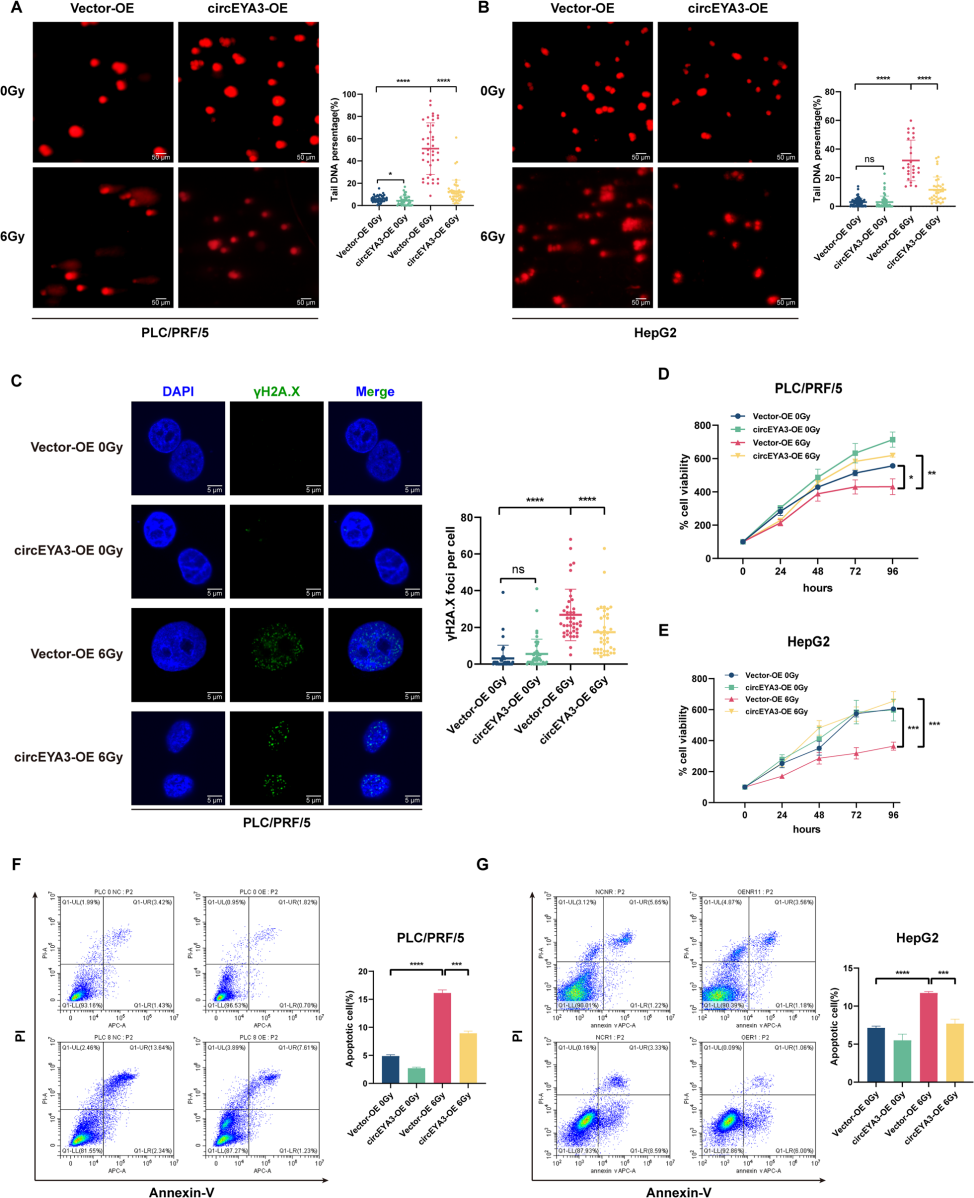
circEYA3 protects HCC cells from DNA damage, proliferation inhibition, and apoptosis induced by external irradiation. **A**, **B** Comet assays were performed to observe DNA damage caused by external irradiation with or without overexpression of circEYA3. **C** Immunofluorescence experiments were conducted to observe γH2A.X foci in PLC/PRF/5 cells with or without external radiation. **D**, **E** Cell proliferation was evaluated using the Cell Counting Kit-8 (CCK-8) assay, revealing that overexpression of circEYA3 suppressed the radiosensitivity of PLC/PRF/5 and HepG2 cells. **F**, **G** Flow cytometry was used to detect apoptotic cells after overexpression of circEYA3 with or without X-ray irradiation at 6 Gy

Because of the different modes of external irradiation and ^125^I irradiation, different biological effects may occur. Therefore, we evaluated the role of circEYA3 in response to external irradiation. Similar to ^125^I radiation, X-ray irradiation at 6 Gy caused an increase in comet tailing, which was alleviated by circEYA3 overexpression, indicating that circEYA3 reduced radiation-induced DNA damage (Fig. 3A, B). Correspondingly, compared with the group that was only treated with X-rays, the number of γH2A.X foci in the circEYA3-overexpressed plus X-ray-treated group significantly decreased (Fig. 3C). Therefore, the DNA damage in HCC cells caused by X-ray irradiation was reversed by the overexpression of circEYA3. The CCK-8 assay showed that X-ray irradiation at 6 Gy reduced the viability of HCC cells, which could be reversed by circEYA3 overexpression. However, in the absence of irradiation, the effect of circEYA3 was not significant (Fig. 3D, E). To further explore the radioresistance function of circEYA3, apoptosis of HepG2 and PLC/PFR/5 cells was evaluated by double staining with Annexin V-PI via flow cytometry. The apoptosis rate was increased by X-ray irradiation at 6 Gy, but this effect was alleviated by the overexpression of circEYA3, in both cell lines (Fig. 3F, G). Meanwhile, the role of circEYA3 in HCC radioresistance was validated through Western blot experiments by assessing the protein expression of γH2A.X, caspase-3, and cleaved caspase-3 (Additional file 1: Figure S1). Taken together, these findings indicate that circEYA3 possessed a radioresistance function in HCC cells under external irradiation.

### CircEYA3 inhibits the radiosensitivity of HCC cells by interacting with IGF2BP2 proteins

**Fig. 4:**
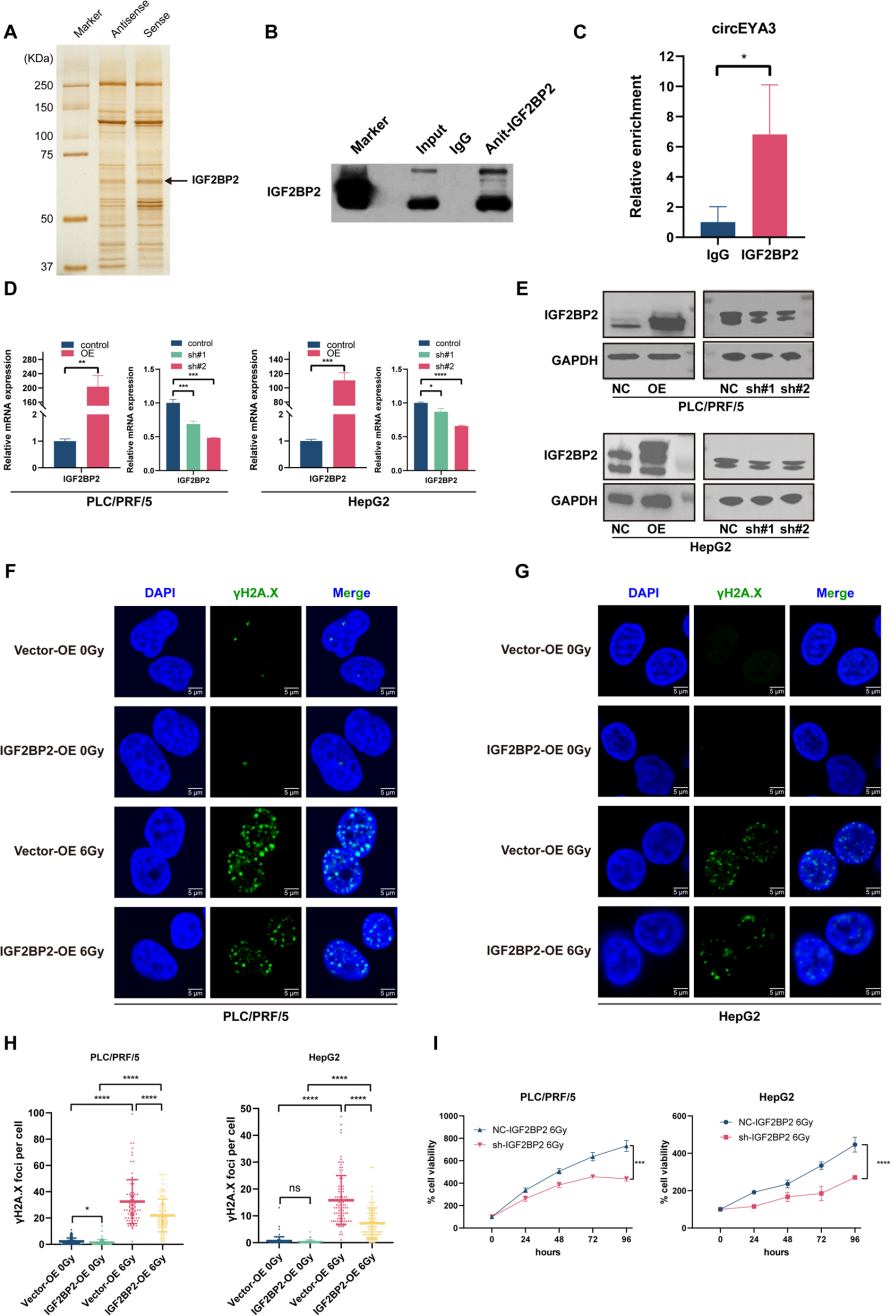
The interaction between circEYA3 and insulin-like growth factor 2 mRNA-binding protein 2 (IGF2BP2) and the function of IGF2BP2 in radiation resistance of HCC. **A**, **B** RNA pulldown assays revealed the interaction between circEYA3 and IGF2BP2 through mass spectrometry and western blotting (WB). **C** RNA immunoprecipitation (RIP) assays showed the association between IGF2BP2 and circEYA3. Immunoglobulin G was used as the negative control. **D**, **E** qRT-PCR and WB assays were used to validate the efficiency of overexpression and knockdown of IGF2BP2. **F–H** γH2A.X immunofluorescence assay was performed to reveal the radiation resistance effect of IGF2BP2. **I** CCK-8 assays showed that the knockdown of IGF2BP2 increased the radiosensitivity of PLC/PRF/5 and HepG2 cell lines

CircRNAs perform their biological functions by interacting with RNA-binding proteins (RBPs) [19]. Given that circEYA3 was mainly located in the cytoplasm, we conducted an RNA pulldown assay to explore its protein-binding role using probes targeting the circEYA3 back-spliced sequence. After silver staining and mass spectrometry analysis, a major differential band was identified as the IGF2BP2 protein (Fig. 4A), which was further validated by WB (Fig. 4B). Subsequently, the interaction between endogenous IGF2BP2 and circEYA3 was confirmed by probing the immunoprecipitates with an anti-IGF2BP2 antibody and the RIP assay (Fig. 4C). However, the overexpression of circEYA3 did not alter IGF2BP2 expression at either the mRNA or protein levels (Fig. S2A, B). Taken together, these findings indicated that circEYA3 interacts with IGF2BP2.

IGF2BP2 has been proven to play an oncogenic role and its expression is associated with poor prognosis in various cancers [20]. However, the role of IGF2BP2 in tumor radiosensitivity remains unclear. First, we constructed knockdown plasmids against IGF2BP2 for validation and selected the sh#2 knockdown plasmid for subsequent experiments. Similarly, the IGF2BP2 plasmid was confirmed to be overexpressed (Fig. 4D, E). The IF assay showed that under non-irradiation conditions, the effect of IGF2BP2 on the γH2A.X foci was negligible. However, with X-ray irradiation of 6 Gy, the increase in γH2A.X foci induced by X-rays was alleviated by the overexpression of IGF2BP2 (Fig. 4F, G, H). The CCK-8 assay also indicated that the knockdown of IGF2BP2 intensified the inhibition of HCC cell proliferation caused by irradiation (Fig. 4I). These data suggest that IGF2BP2 promotes radiation resistance in HCC cells.

**Fig. 5:**
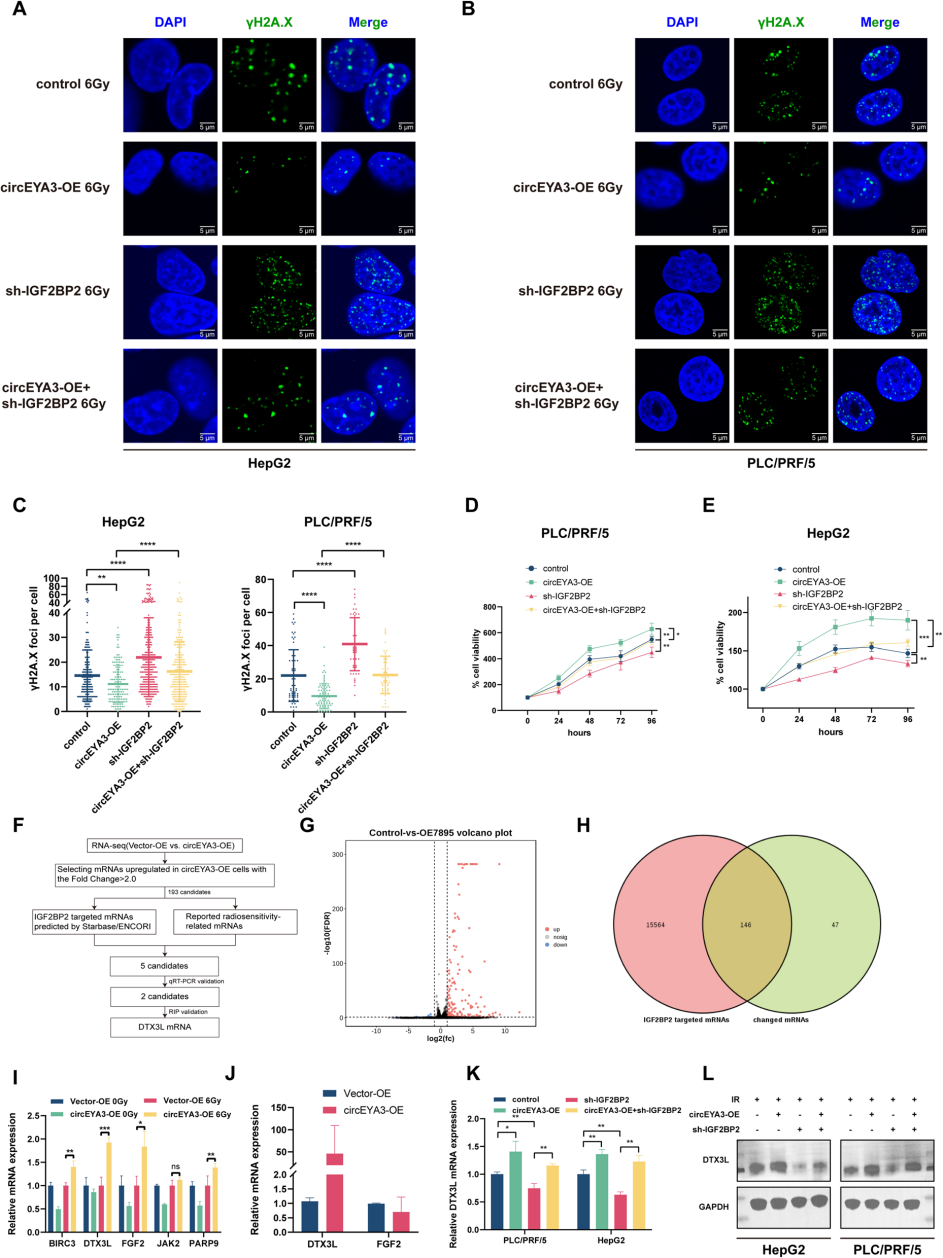
CircEYA3 stabilized *DTX3L* mRNA by interacting with IGF2BP2, thereby increasing the radioresistance of HCC. **A-C** γH2A.X foci were analyzed in circEYA3-overexpressed HCC cells after transfection with short hairpin RNA (sh)-IGF2BP2 or sh-control plasmids. **D**, **E** CCK-8 assays indicated that the radioresistance effect of circEYA3 was rescued by knockdown of IGF2BP2. **F** Downstream mRNA screening process. **G** The volcano map of differentially expressed genes in circEYA3-overexpressed (OE) and Vector-OE cells. **H** Venn diagram showing IGF2BP2-binding mRNA as predicted by starBase/The Encyclopedia of RNA Interactomes database in differentially expressed genes. **I** Relative expression of five candidates after overexpression of circEYA3 with irradiation was detected through qRT-PCR. **J** RIP assay showed that the overexpression of circEYA3 promoted the interaction between IGF2BP2 and *DTX3L* mRNA, but not *FGF2* mRNA. **K**, **L** The expression levels were measured in circEYA3-overexpressed HCC cells after transfection with sh-IGF2BP2 or sh-control plasmids and irradiation using qRT-PCR and WB

Next, we investigated whether circEYA3 reduced the radiosensitivity of HCC cells by interacting with IGF2BP2. As mentioned above, overexpression of circEYA3 reduced the radiation-induced increase in γH2A.X foci, whereas knockdown of IGF2BP2 rescued this phenomenon in both HepG2 and PLC/PRF/5 cell lines (Fig. 5A-C). Similar to these findings, the CCK-8 assay showed that knockdown of IGF2BP2 rescued the reversal effect of circEYA3 overexpression on the radiation-induced decrease in cell viability (Fig. 5D, E). Taken together, these results suggest that the interaction between circEYA3 and IGF2BP2 is a key factor in the radiation resistance of HCC caused by circEYA3.

### CircEYA3/IGF2BP2 upregulates DTX3L mRNA expression

**Fig. 6:**
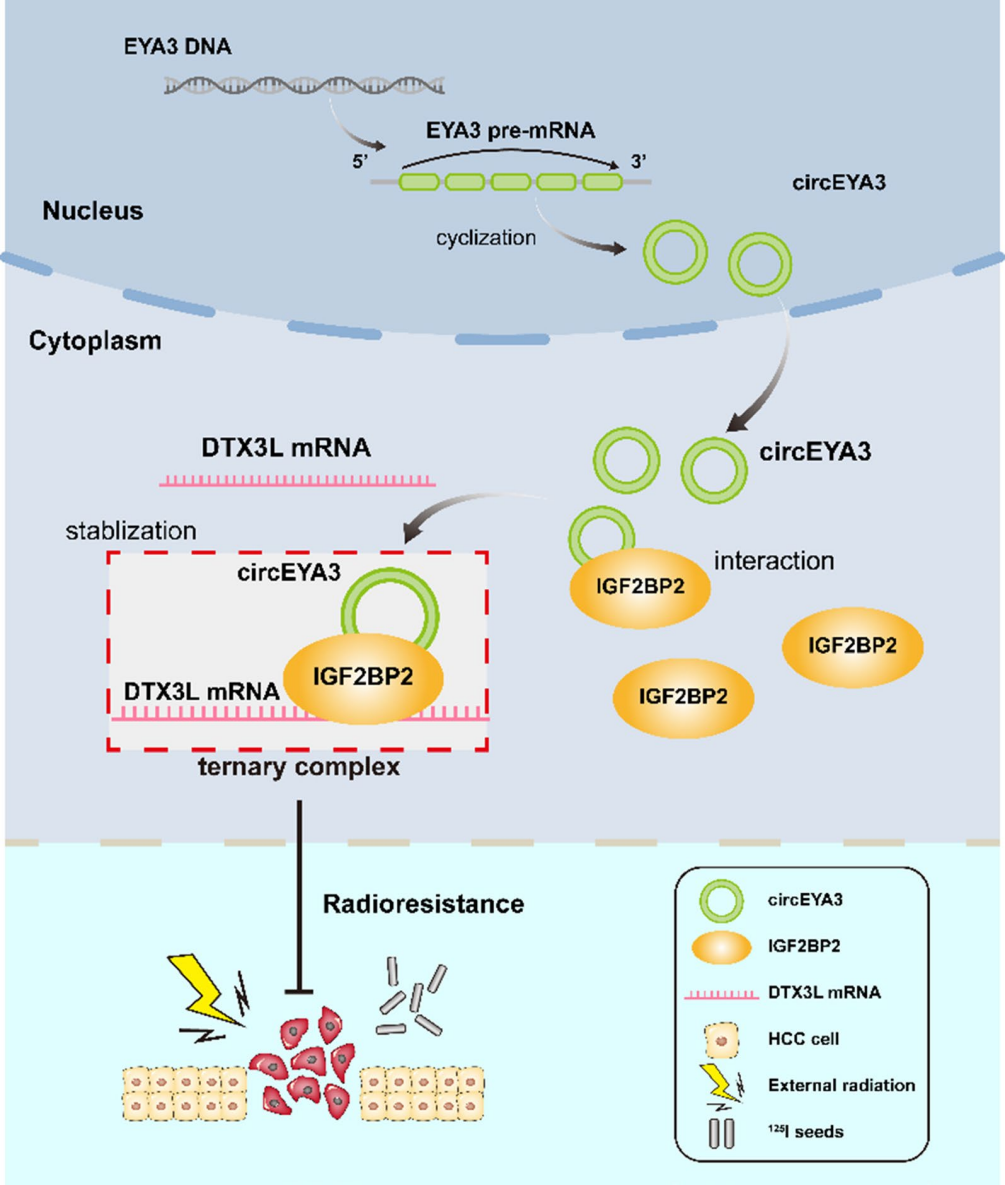
Schematic diagram of the circEYA3-mediated pathway in HCC cells

IGF2BP2 facilitates the stability and storage of its target mRNAs in an m6A-dependent manner, both under normal conditions and in response to stress. This, in turn, exerts an influence on gene expression and translation [21]. Therefore, we hypothesized that circEYA3 may stabilize mRNAs related to irradiation resistance through its interaction with IGF2BP2. To confirm this hypothesis, RNA-seq analysis was performed in PLC/PRF/5 cells with overexpressed circEYA3. In total, 203 genes showed significant differences in expression, including 10 downregulated and 193 upregulated genes (Fig. 5G). After intersecting the 193 upregulated genes with the IGF2BP2-binding mRNAs predicted in the starBase/The Encyclopedia of RNA Interactomes database (https://starbase.sysu.edu.cn/) and excluding genes unrelated to radiosensitivity, five mRNAs were screened (Fig. 5F, H). Next, we performed qRT-PCR for validation, and the results showed that overexpression of circEYA3 upregulated two genes, namely, *DTX3L* and *FGF2*, under irradiation conditions (Fig. 5I). Finally, the RIP assay revealed that with overexpression of circEYA3, the binding of *DTX3L* mRNA and IGF2BP2 protein increased, whereas that of *FGF2* mRNA and IGF2BP2 protein remained unchanged (Fig. 5J). Taken together, these results demonstrate a specific association between circEYA3/IGF2BP2 and *DTX3L* mRNA. Besides, our analysis of The Cancer Genome Atlas database revealed heightened expression of DTX3L in liver cancer tissues compared to normal tissues (Fig. S3). Further validation experiments showed that circEYA3 upregulated DTX3L at both the mRNA and protein levels, and knockdown of IGF2BP2 rescued this effect (Fig. 5K, L). Therefore, circEYA3 upregulated the expression of *DTX3L* mRNA by interacting with IGF2BP2 (Fig. 6).

## Discussion

Tumor radiotherapy resistance remains a challenge that affects the prognosis and treatment of patients with HCC, whether they receive ^125^I brachytherapy or an external irradiation [22]. With the development of sequencing technology and bioinformatics analysis, circRNA, a type of RNA that used to be considered a transcriptional noise and meaningless aberrant splicing by-product, is now gaining attention [23]. circRNAs are known to be involved in external irradiation resistance of HCC [16, 24, 25]. Unlike the X-rays of external irradiation therapy, the irradiation emitted by ^125^I seeds is continuous γ/X-ray at a low dose rate. Therefore, some studies have compared ^125^I radiation with external radiotherapy irradiation and found that the biological effects of radiation are slightly different [26, 27]. However, to the best of our knowledge, there is currently no study revealing the role of circRNAs in ^125^I seed radiation. In this study, we identified that circEYA3 was significantly upregulated in HCC patients receiving ^125^I seed implantation therapy. Further, circEYA3 can increase the radiation resistance of HCC in vivo and in vitro.

CircRNAs function through various regulatory mechanisms, including acting as miRNA sponges [28], protein scaffolds [29], and peptide-encoding genes [30], and regulate the transcription or translation of their parental genes [31]. In terms of interactions with proteins, circRNAs can alter interactions between proteins, tethering or sequestering proteins, recruiting proteins to chromatin, forming circRNA–protein–mRNA ternary complexes, and translocating or redistributing proteins [32]. In this study, we analyzed circEYA3 interacting with proteins using RNA pulldown experiments. Following silver staining, we found several specific bands with molecular weights of 50–75 kDa, which were identified as IGF2BP2 using mass spectrometry and WB. IGF2BP2 is a critical m^6^A reader that regulates post-transcriptional gene expression and participates in a variety of tumor behaviors, including tumor growth, metastasis, angiogenesis, aerobic glycolysis, immune microenvironment, and drug resistance [33–38]. Here, we confirmed the role of IGF2BP2 in radiation resistance in HCC, as it alleviated DNA damage caused by irradiation. Xu et al. suggested that circRNAs can prevent RBP degradation and upregulate its expression by binding to RBP [15]. However, circEYA3 did not increase the expression of IGF2BP2 at either the mRNA or the protein level. Therefore, we performed RNA-seq analysis in PLC/PRF/5 cells and validated the results via qRT-PCR and RIP assays to identify mRNA that positively correlated with circEYA3 expression and interacted with IGF2BP2. As a result, *DTX3L* mRNA was identified. DTX3L is an E3 ubiquitin-protein ligase, which, in association with poly ADP-ribose polymerase-9 (PARP9), plays a role in DNA damage repair [39, 40]. In eukaryotes, DNA DSB repair mainly involves three pathways: homologous recombination, classical non-homologous end junction (C-NHEJ), and alternative NHEJ or micro-homologous-mediated end junction [41, 42]. Among these, the DTX3L/PARP9 complex is involved in the C-NHEJ pathway, which is the most common DSB repair pathway because of its occurrence throughout the cell cycle and ability to ligate any two DNA ends, regardless of the sequence [43]. A study on DTX3L/PARP9 demonstrated that the DTX3L/PARP9 complex has ubiquitin E3 and ADP-ribosyltransferase activities and repairs laser-induced DNA damage identified by γH2A.X foci staining via ubiquitination [44]. In our study, we found that circEYA3 overexpression upregulated the overall expression level of *DTX3L* mRNA, as well as the level of *DTX3L* mRNA binding to IGF2BP2. Given that IGF2BP2 stabilizes mRNA, we speculated that circEYA3 increases the level of *DTX3L* mRNA by promoting the interaction between IGF2BP2 and *DTX3L* mRNA. WB experiments confirmed that the changes in *DTX3L* mRNA could indeed be successfully transformed to the protein level, thereby playing a repair role in radiation-induced DNA damage.

This study had a few limitations. First, the traditional treatment mode of HCC limits the acquisition of tumor tissue samples from patients. ^125^I brachytherapy is generally applied to recurrent HCC after comprehensive treatment or to metastatic lymph nodes [8, 45–47]. At this point, patients are no longer suitable for undergoing liver resection again for tumor sample collection. Second, in addition to protein scaffolds, circRNAs participate in other biological processes. Whether circEYA3 increases the radioresistance of HCC through miRNA sponges or peptide translation requires further investigation. Finally, the specific sequence of IGF2BP2 that binds to circEYA3 and *DTX3L* mRNA, as well as whether it is mediated through the m^6^A pathway, remains to be studied.

## Conclusions

In conclusion, our study firstly demonstrated that circEYA3 was significantly upregulated in HCC patients who received ^125^I seed implantation therapy. Overexpression of circEYA3 increased radioresistance to ^125^I brachytherapy and external irradiation. Mechanistically, circEYA3 directly binds to IGF2BP2 and enhances its function of stabilizing *DTX3L* mRNA, thereby repairing radiation-induced DNA damage. Our study unveils the mechanisms of hepatocellular carcinoma (HCC) resistance to ^125^I seed and external radiation from the perspective of circRNA. This provides a novel indicator for predicting the radiosensitivity of HCC and offers new targets and intervention options for radiotherapy in HCC. Future endeavors will prioritize larger clinical sample sizes and the development of potential pharmaceutical interventions.

### Supplementary Information

**Additional file1: Table S1.**The clinical information of patients undergoing RNA sequencing. **Table S2**.Key primers and oligos. **Table S3. **The top 10 significant circRNAs related to radiosensitivity. **Figure S1. **The role of circEYA3 in HCC radioresistance was validated through Western blot experiments. **Figure S2. **Western blot and qPCR experiments indicated that the overexpression of circEYA3 did not affect the expression of IGF2BP2 at the protein and mRNA levels. **Figure S3. **Bioinformatics analysis targeting the TCGA database revealed elevated expression of DTX3L mRNA in liver cancer compared to normal tissues.

## Data Availability

All data generated or analyzed during this study are included in this article or in the supplementary information files.

## Abbreviations

CCK-8: Cell Counting Kit-8
circRNA: Circular RNA
DTX3L: Deltex E3 ubiquitin ligase 3L
DSBs: DNA double-strand breaks
EYA3: Eyes absent homolog 3
FISH: Fluorescence in situ hybridization
GAPDH: Glyceraldehyde-3-phosphate dehydrogenase
HCC: Hepatocellular carcinoma
IF: Immunofluorescence
IGF2BP2: Insulin-like growth factor 2 mRNA-binding protein 2
PBS: Phosphate-buffered saline
PARP9: Poly ADP-ribose polymerase-9
PLC: Primary liver cancer
PI: Propidium iodide
qRT-PCR: Quantitative real-time PCR
RBPs: RNA-binding proteins
RIP: RNA immunoprecipitation
RNA-seq: RNA sequencing
shRNAs: Short hairpin RNAs
SCGE: Single cell gel electrophoresis assay
WB: Western blotting
