# Supplementary material for: Circular RNA circEYA3 promotes the radiation resistance of hepatocellular carcinoma via the IGF2BP2/DTX3L axis
Source: Cancer Cell Int. 2023 Dec 2;23:308. doi: 10.1186/s12935-023-03168-2 (PMC10693171; doi:10.1186/s12935-023-03168-2)
Supplement: Supplementary file 1 — Additional file1: Table S1.The clinical information of patients undergoing RNA sequencing. Table S2.Key primers and oligos. Table S3. The top 10 significant circRNAs related to radiosensitivity. Figure S1. The role of circEYA3 in HCC radioresistance was validated through Western blot experiments. Figure S2. Western blot and qPCR experiments indicated that the overexpression of circEYA3 did not affect the expression of IGF2BP2 at the protein and mRNA levels. Figure S3. Bioinformatics analysis targeting the TCGA database revealed elevated expression of DTX3L mRNA in liver cancer compared to normal tissues. [file 12935_2023_3168_MOESM1_ESM.docx]

**Circular RNA circEYA3 promotes the radiation resistance of hepatocellular carcinoma via the IGF2BP2/*DTX3L* axis**

Pan Hu, Letao Lin, Tao Huang, Zhenyu Li, Meigui Xiao, Huanqing Guo, Guanyu Chen, Dengyao Liu, Miaola Ke, Hongbo Shan, Fujun Zhang, Yanling Zhang

**Additional Information**

| Patient No. | Age | Gender | BCLC stage | AFP (ng/ml) | Tumor size (cm) | Number of tumors | Vascular invasion |
| --- | --- | --- | --- | --- | --- | --- | --- |
| 1 | 39 | Male | C | 31.72 | 1.7 | Single | Yes |
| 2 | 60 | Female | C | 175.9 | 3.3 | Single | No |
| 3 | 44 | Male | A | 538.4 | 1.0 | Single | No |
| 4 | 55 | Female | A | 379.7 | 2.1 | Single | No |
| 5 | 58 | Male | B | 3.87 | 1.5 | Multiple | No |
| 6 | 69 | Male | C | 5.40 | 1.8 | Single | No |

**Table S1** The clinical information of patients undergoing RNA sequencing

**Table S2** Key primers and oligos

| Items | Sequences |
| --- | --- |
| circEYA3 qRT-PCR primers | F: CACATATTCTCTCAGTTCCTGT  R: CAGCAGTTTTCACAATCAAAAGG |
| EYA3 qRT-PCR primers | F: GCAGTAGCCAGCATCTCAAACC  R: GTCTGACCTGTGACTCCAAAGC |
| IGF2BP2 qRT-PCR primers | F: GTTGGTGCCATCATCGGAAAGG  R: TGGATGGTGACAGGCTTCTCTG |
| BIRC3 qRT-PCR primers | F: GCTTTTGCTGTGATGGTGGACTC  R: CTTGACGGATGAACTCCTGTCC |
| DTX3L qRT-PCR primers | F: CCAGGTTATGAGTCCTTTGGCAC  R: TGCAGTTCGCTGTATTCCAGGG |
| FGF2 qRT-PCR primers | F: AGCGGCTGTACTGCAAAAACGG  R: CCTTTGATAGACACAACTCCTCTC |
| JAK2 qRT-PCR primers | F: CCAGATGGAAACTGTTCGCTCAG  R: GAGGTTGGTACATCAGAAACACC |
| PARP9 qRT-PCR primers | F: GGCAAAGAGGTCCAAGATGCTG  R: GCCTCACACATCTCTTCCACGT |
| U6 qRT-PCR primers | F: CTCGCTTCGGCAGCACA  R: AACGCTTCACGAATTTGCGT |
| circEYA3 FISH detecting probe | 5' TTCACAATCAAAAGGAGGTAGTC 3' |
| circEYA3 overexpression plasmid | F: GAATTCTAATACTTTCAGATTGTGAAAACTGCTGAGAGA  R: GGATCCAGTTGTTCTTACCAAAAGGAGGTAGTCCATACG |
| sh-IGF2BP2#1 | AGCGCAAGATCAGGGAAATTG |
| sh-IGF2BP2#2 | AGTGAAGCTGGAAGCGCATAT |

**Table S3** The top 10 significant circRNAs related to radiosensitivity

| AccID | circRNA | GeneName | Label |
| --- | --- | --- | --- |
| chr11_108229323_108227595_+1728 | hsa_circ_0024193 | ATM | Down |
| chr7_99338285_99333325_+4960 | hsa_circ_0002425 | ARPC1AA | Up |
| chr3_197875775_197865423_+10352 | hsa_circ_0008351 | LRCH3 | Down |
| chr6_130184623_130184103_-520 | hsa_circ_0008833 | SAMD3 | Down |
| chr1_28058094_28035544_-22550 | hsa_circ_0007895 | EYA3 | Up |
| chr2_9350895_9344532_+6363 | hsa_circ_0052525 | ASAP2 | Down |
| chr4_2951245_2949934_-1311 | hsa_circ_0006737 | NOP14 | Down |
| chr3_71699708_71690010_-9698 | hsa_circ_00665358 | EIF4E3 | Down |
| chr2_214797117_214767482_-29635 | hsa_circ_0058051 | BARD1 | Down |
| chr3_49325845_49324702_-1143 | hsa_circ_0004609 | USP4 | Down |

**
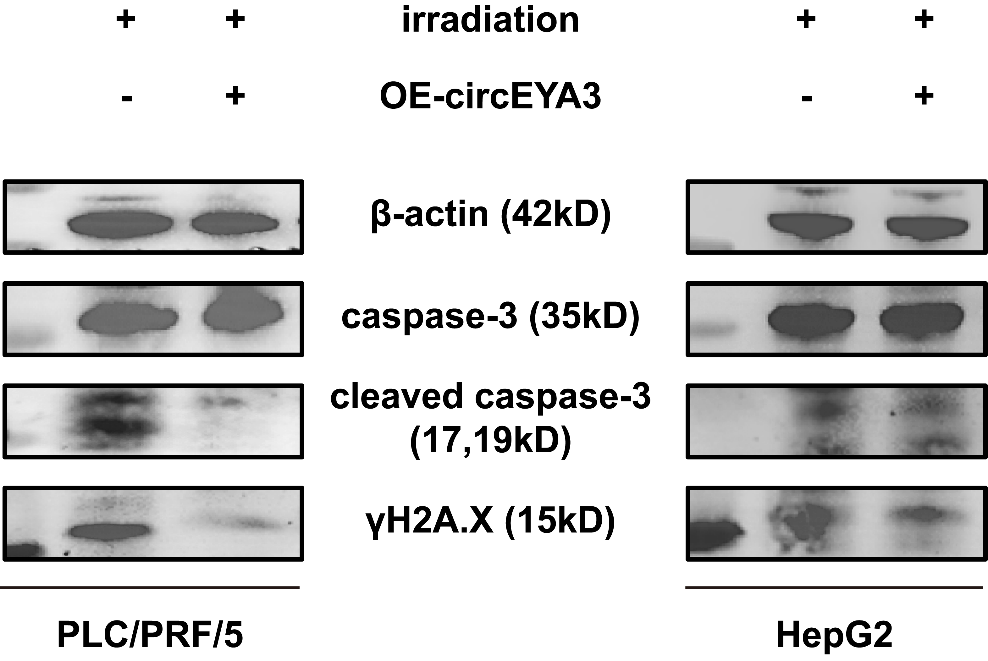
**

**Additional Figure S1** The role of circEYA3 in HCC radioresistance was validated through Western blot experiments.


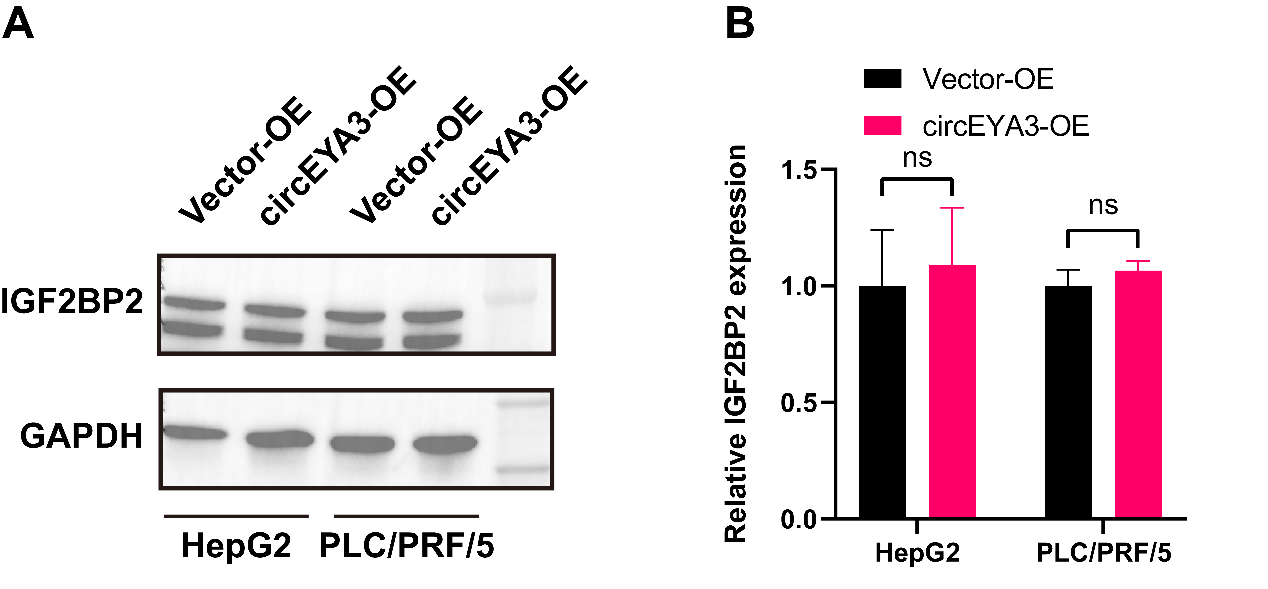


**Additional Figure S2** Western blot and qPCR experiments indicated that the overexpression of circEYA3 did not affect the expression of IGF2BP2 at the protein and mRNA levels.


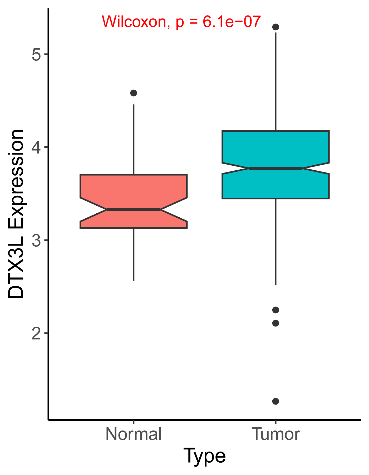


**Additional Figure S3** Bioinformatics analysis targeting the TCGA database revealed elevated expression of DTX3L mRNA in liver cancer compared to normal tissues.
